# Supplementary material for: Secular Trends in Incidence of Esophageal Cancer in Taiwan from 1985 to 2019: An Age-Period-Cohort Analysis
Source: Cancers (Basel). 2022 Nov 27;14(23):5844. doi: 10.3390/cancers14235844 (PMC9741308; doi:10.3390/cancers14235844)
Supplement: Supplementary file 1 [file cancers-14-05844-s001.zip › cancers-2004216-supplementary.pdf]

Supplementary Materials: Secular trends of esophageal cancer in Taiwan from 1985 to 2019: An age−period−cohort analysis

Min−Chen Tsai, Yu−Ching Chou, Yu−Kwang Lee, Wan−Lun Hsu, Chin−Sheng Tang, Shiow−Ying Chen, Shih−Pei Huang, Yong−Chen Chen and Jang−Ming Lee

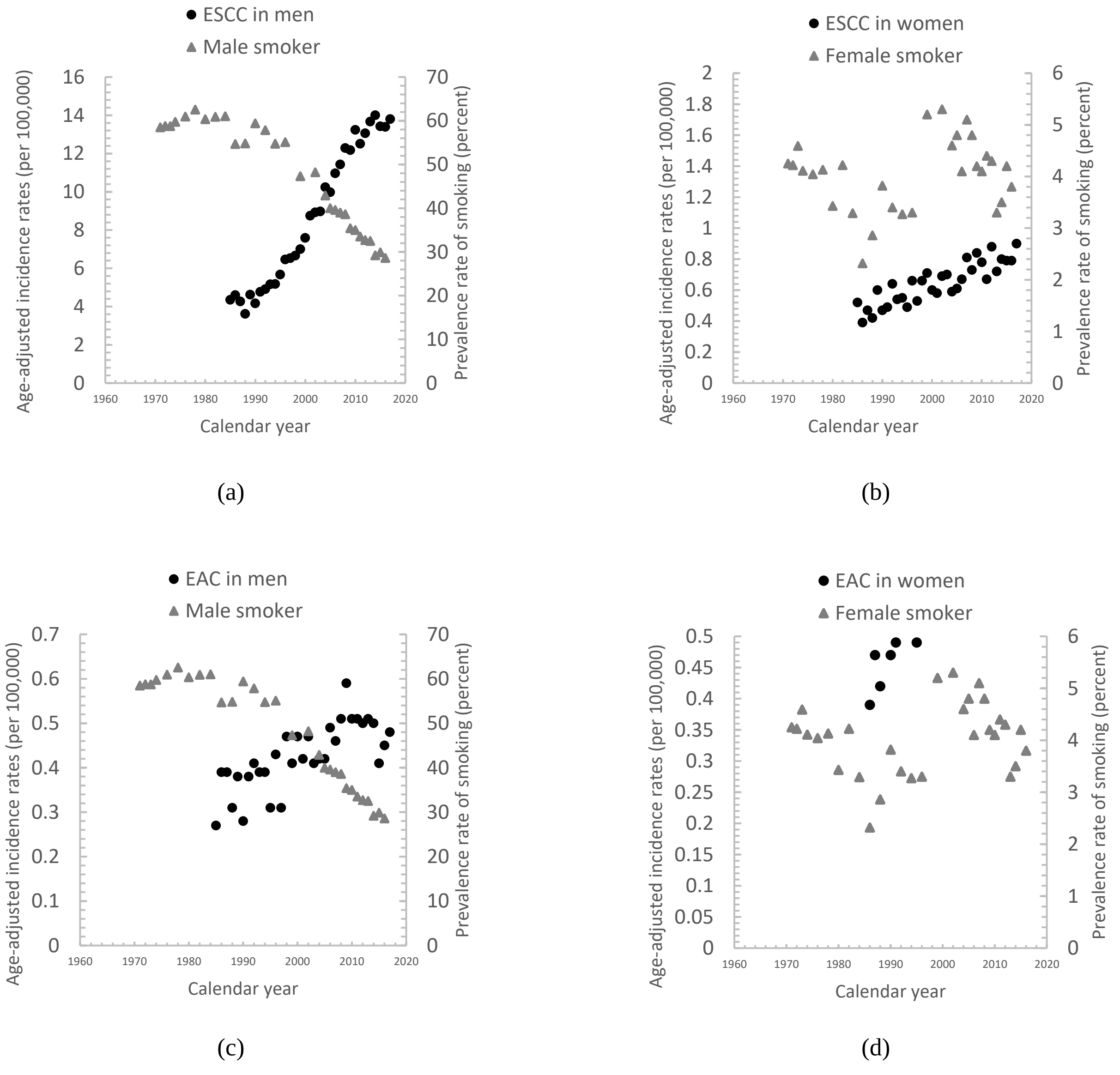

Figure S1. Prevalence of smoking in 1971−1982 and incidence of esophageal squamous cell carcinoma (ESCC) and adenocarcinoma (EAC) in 2006−2017. (a) ESCC in men; (b) ESCC in women; (c) EAC in men; (d) EAC in women.

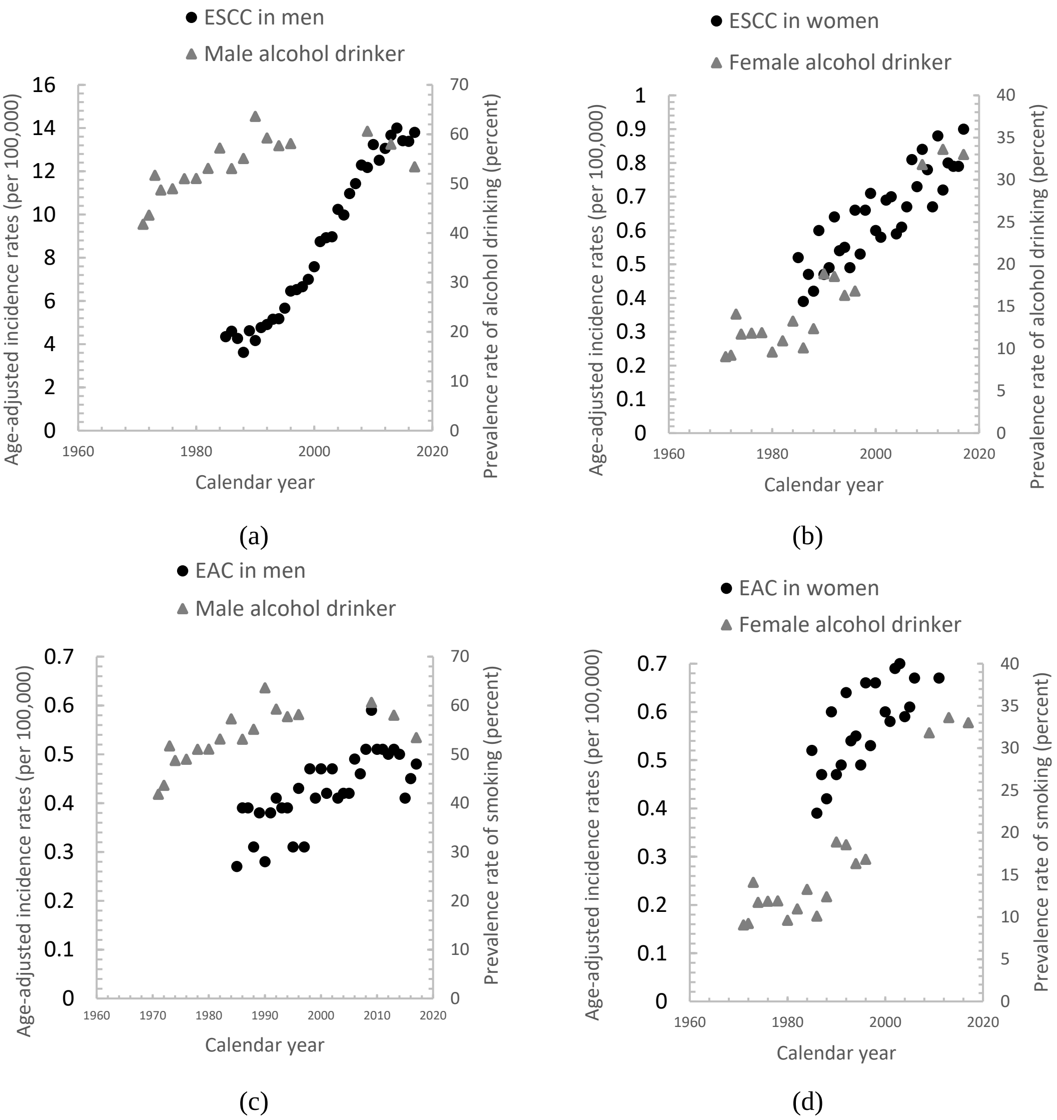

Figure S2. Prevalence of alcohol consumption in 1971−1982 and incidence of esophageal squamous cell carcinoma (ESCC) and adenocarcinoma (EAC) in 2006−2017. a: ESCC in men; b: ESCC in women; c: EAC in men; d: EAC in women.

Table S1. Values of age−adjusted incidence rates of esophageal cancer (EC), esophageal squamous cell carcinoma (ESCC), and esophageal adenocarcinoma (EAC) by sex in Taiwan for the period 1985−2019.

Year	Age−adjusted Incidence Rates Per 100,000 (95% CI)					
	EC		ESCC		EAC	
	Men	Women	Men	Women	Men	Women
1985−1989	5.82 (5.06, 6.58)	0.70 (0.43, 0.97)	4.29 (3.65, 4.93)	0.48 (0.26, 0.70)	0.35 (0.16, 0.53)	0.06 (−0.01, 0.14)
1990−1994	6.22 (5.49, 6.95)	0.82 (0.55, 1.09)	4.85 (4.22, 5.49)	0.54 (0.33, 0.75)	0.37 (0.19, 0.55)	0.07 (0.00, 0.13)
1995−1999	7.75 (7.00, 8.53)	0.86 (0.61, 1.12)	6.47 (5.78, 7.17)	0.61 (0.40, 0.82)	0.39 (0.23, 0.56)	0.08 (0.02, 0.14)
2000−2004	10.10 (9.29, 10.92)	0.87 (0.64, 1.09)	8.92 (8.17, 9.68)	0.63 (0.44, 0.82)	0.44 (0.27, 0.61)	0.11 (0.03, 0.19)
2005−2009	12.55 (11.70, 13.40)	0.90 (0.68, 1.13)	11.4 (10.59, 12.20)	0.74 (0.53, 0.94)	0.49 (0.33, 0.66)	0.08 (0.02, 0.14)
2010−2014	14.36 (13.25, 15.46)	0.91 (0.63, 1.20)	13.3 (12.25, 14.36)	0.77 (0.51, 1.03)	0.5 (0.29, 0.72)	0.07 (0.00, 0.15)
2015−2019	14.53 (13.72, 15.34)	0.97 (0.75, 1.19)	13.49 (12.73, 14.26)	0.82 (0.63, 1.02)	0.47 (0.31, 0.64)	0.06 (0.01, 0.11)

Table S2. Annual percent change (APC) and average annual percent change (AAPC) in ESCC over time, by sex and age.

Characteristics	Joinpoint Segment Year Start	Joinpoint Segment Year End	APC (95% CI)		*p*−Value	AAPC (95% CI)		*p*−Value
men								
40−44	1985	2009	11.5	(11~12.1)	< 0.001	7.1	(6.9~7.3)	< 0.001
	2010	2019	−1.4	(−2.1~−0.7)	0.01			
45−49	1985	2009	10.8	(9.3~12.3)	<0.01	7.7	(7.1~8.2)	< 0.001
	2010	2019	1.6	(−0.2~3.5)	0.06			
50−54	1985	2019	5.7	(4.1~7.4)	< 0.001			
55−59	1985	2009	5.7	(3.1~8.4)	<0.05	5.0	(3.9~6)	< 0.001
	2010	2019	3.5	(−0.8~8)	0.08			
60−64	1985	2019	3.5	(2.6~4.3)	<0.001			
65−69	1985	2019	2.5	(1.7~3.4)	<0.01			
70−74	1985	2019	1.5	(1.1~2)	<0.001			
75−79	1985	2019	1.0	(0.7~1.4)	<0.01			
women								
40−44	1985	2019	3.8	(−0.7−8.6)	0.08			
45−49	1985	2019	5.6	(1.8−9.6)	0.01			
50−54	1985	2019	5.0	(3.7−6.4)	< 0.001			
55−59	1985	1999	−4.3	(−22.5−18.2)	0.47	1.9	(−1.6~5.5)	0.29
	2000	2019	5.1	(0.5−10)	0.04			
60−64	1985	2019	1.4	(−0.1−2.9)	0.07			
65−69	1985	2019	−0.1	(−1.1−1)	0.86			
70−74	1985	2019	0.0	(−1.9−2)	0.96			
75−79	1985	1999	7.0	(−0.8−15.4)	0.06	0.1	(−1.1~1.3)	0.88
	2000	2019	−3.2	(−4.6−−1.8)	0.01			

Table S3. Annual percent change (APC) and average annual percent change (AAPC) in EAC over time, by sex and age.

Characteristics	Joinpoint Segment Year Start	Joinpoint Segment Year End	APC (95% CI)		*p*–Value	AAPC (95% CI)	*p*–Value
men							
40–44	1985	2019	3.6	(0.5~6.8)	0.03		
45–49	1985	2019	5.2	(0.3~10.3)	0.04		
50–54	1985	2019	3.5	(1.1~5.9)	0.01		
55–59	1985	2019	1.8	(−0.9~4.7)	0.15		
60–64	1985	2019	1.6	(0.5~2.8)	0.01		
65–69	1985	2019	0.0	(−1.3~1.3)	1.00		
70–74	1985	2009	1.8	(−3.6~7.5)	0.29	−0.4 (−3.7~2.9)	0.81
	2010	2019	−4.7	(−1.1~15.2)	0.39		
75–79	1985	2019	−1.7	(−3.6~0.3)	0.08		
women							
40–44	–						
45–49	–						
50–54	–						
55–59	–						
60–64	1985	2019	−2.3	(−8~3.8)	0.37		
65–69	1985	2019	−0.1	(−1.9~1.7)	0.87		
70–74	1985	2019	−1.1	(−5.0~3.0)	0.51		
75–79	1985	1999	11.1	(−26.8~68.6)	0.39	−0.7 (−7.1~6.2)	0.84
	2000	2019	−6.1	(−13~1.3)	0.07		
